# Predicting patterns of service utilization within children’s mental health agencies

**DOI:** 10.1186/s12913-019-4842-2

**Published:** 2019-12-23

**Authors:** Graham J. Reid, Shannon L. Stewart, Melanie Barwick, Jeffrey Carter, Alan Leschied, Richard W. J. Neufeld, Jeff St. Pierre, Juliana I. Tobon, Evelyn Vingilis, Gregory S. Zaric

**Affiliations:** 1 Departments of Psychology, Family Medicine, and Paediatrics, The University of Western Ontario, Westminster Hall Room 319E, London, ON N6A 3K7 Canada; 2 Children’s Health and Therapeutics, Children’s Health Research Institute, London, Canada; 3 Faculty of Education, The University of Western Ontario, London, Canada; 4 Research Institute, The Hospital for Sick Children, Toronto, Canada; 5 Department of Psychiatry, University of Toronto, Toronto, Canada; 6 The Dalla Lana School of Public Health, University of Toronto, Toronto, Canada; 7 Quality Improvement, Vanier Children’s Services, London, Canada; 8 Departments of Psychology and Psychiatry, The University of Western Ontario, London, Canada; 9 Departments of Psychology, Psychiatry, and Neuroscience, The University of Western Ontario, London, Canada; 10 Child and Parent Resource Institute, London, Canada; 11 Department of Psychology, The University of Western Ontario, London, Canada; 12 St. Michael’s Hospital Academic Family Health Team, Toronto, Canada; 13 Departments of Family Medicine and Epidemiology and Biostatistics, The University of Western Ontario, London, Canada; 14 Ivey Business School, The University of Western Ontario, London, Canada; 15 Department of Epidemiology and Biostatistics, The University of Western Ontario, London, Canada

**Keywords:** Patterns of service use, Mental health, Children, Mental health services

## Abstract

**Background:**

Some children with mental health (MH) problems have been found to receive ongoing care, either continuously or episodically. We sought to replicate patterns of MH service use over extended time periods, and test predictors of these patterns.

**Methods:**

Latent class analyses were applied to 4 years of visit data from five MH agencies and nearly 6000 children, 4- to 13-years-old at their first visit.

**Results:**

Five patterns of service use were identified, replicating previous findings. Overall, 14% of cases had two or more episodes of care and 23% were involved for more than 2 years. Most children (53%) were seen for just a few visits within a few months. Two patterns represented cases with two or more episodes of care spanning multiple years. In the two remaining patterns, children tended to have just one episode of care, but the number of sessions and length of involvement varied. Using discriminant function analyses, we were able to predict with just over 50% accuracy children’s pattern of service use. Severe externalizing behaviors, high impairment, and high family burden predicted service use patterns with long durations of involvement and frequent visits.

**Conclusions:**

Optimal treatment approaches for children seen for repeated episodes of care or for care lasting multiple years need to be developed. Children with the highest level of need (severe pathology, impairment, and burden) are probably best served by providing high intensity services at the start of care.

## Predicting patterns of service utilization within children’s mental health agencies

The natural history of mental health (MH) problems suggests that a sizable percentage of children with MH problems might need ongoing care, either continuously or episodically. There is extensive evidence of the continuity from childhood to adolescence for both externalizing [i.e., attention deficit hyperactivity (ADHD), oppositional defiant (ODD), and conduct disorders] and internalizing problems (i.e., anxiety, depression; [1, 2]). Many children have either ongoing MH problems or experience recurrent episodes of MH problems. Ongoing problems with depression occur for 10–18% and for anxiety, 41–66% [3]. Recurrence rates for depression are 50–70% in natural history studies and 15–47% of children have a relapse following treatment [3]. It is common for ADHD to persist for years [4] with 28–60% of children continuing to have ADHD for many years [5–7]. Similarly, about 20% of children have persistent ODD [8] while 50% may have other types of MH problems years later [9].

This presents a problem for how child and youth mental health service (CYMHS) delivery systems can best care for these children. Evidence-based treatments (EBTs) exist for the most common MH problems seen in CYMHS agencies (e.g., [10]). However, we know of no specific protocols outlining how best to care for children who might present for repeated episodes of care (EoC), or for whom treatment lasts years, rather than months. In general, little is known about how children with MH problems use services over extended time periods. We also need to be able to identify predictors of divergent care experiences, as a first step in developing protocols to care for children with ongoing or episodic MH care needs. The current study adds to the few studies (e.g., [11–15]) that have examined service use over multiple years and examines predictors of patterns of service use.

### Patterns of service use over extended time periods

Paucity of research on children’s use of MH services over extended time periods is likely due to challenges conducting such research. A full understanding of service use over extended time periods requires multiple years of the following data, for each episode of care, for all children seen within a CYMHS agency: (a) problem type and severity at the start of services, (b) CYMHS received (e.g., was an EBT received), (c) predictors of treatment engagement and/or effectiveness (e.g., sociodemographics, therapeutic alliance), and (d) problem severity and (e) disposition (e.g., drop out, mutual agreement to end services) at the end of services. It is doubtful that any agency would have such a dataset. For example, in the UK Child Outcomes Research Consortium [16] dataset (> 250,000 cases), “only 24% [of cases] have meaningful outcome data” (p. 300). Prospective studies could obtain a number of the above variables, but recruitment and low participation rates may be a problem [17, 18]. For example, only 50% of youth participated in the Patterns of Care study in the US [18]. Loss to follow-up is a further problem in prospective studies (e.g., [19–21]) or studies recruiting clients after discharge [22]; these rates vary from about 30% [19] to over 90% [22]. In light of these challenges, we used administrative data from CYMHS agencies to examine patterns of service use over multiple years. This provided complete visit data on all children, but limited amounts of clinical information. We used a person-centered approach beginning with the child’s first visit and examining all visits within the next 4 years to better capture how families experienced services over time. When fiscal or calendar years are used (e.g., [23, 24]), children can be at various points in their care within a year, and thus the child/family’s pattern of service use over time is obscured.

Most studies that examined patterns of children’s MH services used periods of 1 year or less [11, 12, 19, 20, 25, 26]. Three studies had longer time frames and examined different aspects of service use: (a) 10–15 year retrospective parent-reports of services [13]; (b) rural-urban differences in service use based on 5 years of administrative data, [14]; (c) changes in service use with wraparound treatment based on 6 years of claims data [15]. The current study adds to the literature by examining all services used by children seen in five CYMHS agencies over a 4-year time period.

Our research team recently completed a study examining the patterns of service use over a 5-year time period for children age 4- to 11-years old at the time of their first visit to one of six CYMHS agencies in Ontario in 2000, − 01, or − 02 [27]. We identified five patterns of service use that were stable across agencies and across time (i.e., intake years 2000–02). For example, the Minimal care group (48% of cases) had the shortest duration of services and fewest visits. Two service use patterns (19% of cases) characterized children who had multiple EoCs; an EoC was defined as a minimum of three visits with at least 6 months in between the end of one EoC and the start of the next [28]. We do not know if these patterns were unique to these agencies or the study time-period. Thus, the first aim of the present study was to see if we could replicate these patterns of service use in a new sample of children and youth.

### Predicting patterns of service use

The ability to identify predictors of divergent care experiences is an important first step in developing better ways to provide CYMH services. Our previous study, however, found few child or family characteristics differentiated patterns of care [27]. Thus, the second aim of the current study was to examine predictors of patterns of service use.

Andersen’s behavioral model of health services utilization framed our choice of predictors [29, 30]. We expected that service utilization would be predicted by predisposing (e.g., sex, age), enabling (e.g., burden of illness), and need (e.g., child psychopathology) factors. There have been few studies examining predictors of service use over extended time periods. Studies of accessing care have found mixed relationships for child age [31–33] and sex [34, 35] as predictors. Greater severity of psychopathology tends to be associated with service use [31, 35], as have higher levels of parental burden [36]. In light of these findings, we hypothesized that more severe child psychopathology and higher family burden would be associated with more intensive patterns of service use; i.e., patterns characterized by longer durations of care, more visits, and repeated EoCs. Descriptive statistics related to service use (e.g., total number of visits, duration of involvement, episodes of care) for each pattern were examined to inform the labelling and interpretation of the patterns.

## Methods

We conducted secondary data analysis of administrative data for 2004–2010 from five CYMHS agencies that: (a) provided services for children and youth (age 5–18 years), and (b) were accredited by Children’s Mental Health Ontario. We purposely selected CYMHS agencies in Ontario that are located in, and serve, both rural and urban populations, and that were willing to partner with us. Access to publicly-funded CYMH care in Ontario is based on need, and does not depend on access to insurance coverage and/or the ability to pay, or require a diagnosis, and is available to any child or youth within the catchment area of the agency. In Ontario, specialized CYMHS for 0–18 year olds has been delivered by agencies funded by a ministry other than health [37] and has operated separately from MH care provided by family physicians, pediatricians and psychiatrists, which is also free and covered under provincial healthcare plans [38, 39].

### Participant selection

Inclusion criteria were as follows: [1] the first face-to-face visit to the agency occurred in 2004–2006 (operationally defined as having no face-to-face visit in the previous 18 months); and [2] clients were between the ages of 5 to 13 years at the time of the first face-to-face visit. We used an upper age of 13 years to ensure that it was possible for the oldest children in the study to receive services 4 years after their first visit before potentially aging-out and/or being transferred to adult services at 18 years of age. We used a lower age of 5 years because the infant and preschool version of the Brief Child and Family Phone Interview (BCFPI; [40, 41]) did not yet exist [42]. We examined 4 years of data following the child’s first visit to the agency since most differences between service use patterns in our previous study [27] were evident within 4 years, with few additional differences evident in year 5. There were 12,643 cases that met the first criterion; the 24% (*n* = 3099) of cases who were less than age 5 and the 21% (*n* = 2711) who were older than 13 were removed.

Exclusion criteria were: [1] children with (a) a pervasive developmental disorder (i.e., Asperger’s syndrome, autism), or (b) a developmental disability (e.g., Down syndrome), or children who received services from a program specializing in these disorders at the agency at any point within the study window (*n* = 841). These cases were excluded, as children with these problems are known to need ongoing care and treatment models sensitive to ongoing care needs already exist [2, 43–45]. Clients who had an EoC within the previous 18 months were also excluded (*n* = 360); this meant that children were clearly starting a new EoC. Other studies have used a much shorter period of time (e.g., 12 weeks [46]; first 60 days of the year [25]) for excluding cases, or have not specified if children were excluded based on previous visits (e.g., [47, 48]).

### Administrative data

Raw data in electronic format were received from each participating CYMHS agency including: child date of birth, sex, and visit data [e.g., date, type of contact (e.g., telephone, in-person, in-home visit)]. Only face-to-face visits were included. Telephone contacts were excluded because it was unclear whether these contacts were for administrative purposes (e.g., rescheduling appointments), or if treatment was provided. All non-direct contact appointments were also excluded (e.g., scheduling, report writing).

To examine patterns of service use, the first aim of the study, the date of each child’s first visit was set to a visit in ‘month 1’. Visit data were then recoded to indicate whether or not a child had been seen within each month in the 48 months (4 years) following the child’s first in-person visit.

For descriptive purposes, the total number of visits and duration of involvement (i.e., time between first and last visit), and percentage of cases with involvement lasting more than 2 years were calculated. The volume of services used for each pattern was calculated as a percentage as follows: the number of visits was summed across clients and agencies within each of the five patterns, and divided by the sum of all visits for all clients and agencies. To examine the intensity of service use, the total number of visits per year was examined. Visits were also organized into EoCs, using the previously published definition of an EoC [28].

#### Predictor variables

Child date of birth and sex were obtained from the administrative data. Date of birth was used to compute child’s age at the time of the first visit to the agency.

#### Brief child and parent phone interview (BCFPI)

The BCFPI assesses child psychopathology and other factors known to influence treatment engagement (e.g., impact of illness on the family), and was a mandated intake measure at Ontario CYMHS agencies between 2001 and 2015 [49]; due to a shift in government policy, its use was not mandated after 2015 (M. Barwick, personal communication, Nov 2019). The BCFPI assessment that was closest to the date of the child’s first in-person visit was used; i.e., within 8 months before the first visit or 1 month after. Four composite scales were calculated by combining multiple subscales [41]. Externalizing Behavior - 18 items related to regulating attention and impulsivity, cooperativeness, and conduct; Internalizing Behavior - 18 items related to managing anxiety and mood, as well as separation from parents. The Global Child Functioning scale (hereafter Child Impairment) is an index of impairment that measures social participation, quality of relationships, and school performance and achievement. Finally, the Global Family Situation scale (hereafter Family Burden), which reflects the burden of illness for the family, includes items related to the impact of the child’s problems on family activities (i.e., external family functioning) and family comfort (i.e., internal family functioning). The BCFPI psychopathology scales have good internal consistency (α ≥ 0.74) and test-retest reliability (*r* ≥ 0.54; [50]). In clinical samples, convergent validity include correlations with symptom counts on a diagnostic interview (*r* = 0.68–0.78); and confirmatory factor analyses provide support for construct validity [51]. Using age- and sex-based norms, raw scores were converted to T-scores based on comparisons to the general population. Groups were formed based on T-scores above or below the clinical cut-off (T-score ≥ 65; 93rd percentile).

A missing values analysis (conducted in SPSS v24 [52]) was completed on available BCFPI data. Missing values ranged from 0.3% (Externalizing Behavior) to 12.4% (Family Burden). Little’s MCAR test indicated that these missing values were not random [χ^2^(794) = 1073.811, *p* < 0.001]. Missing BCFPI values were imputed using an Expectation-Maximization algorithm, which iteratively verifies imputed values against other variables to reach the most likely value, preserving the relationships between variables.

### Data analysis

#### Examining patterns of service use: latent class analysis

Multi-level latent class analysis (LCA; Latent Gold v. 4.5) was applied to data across all agencies to determine patterns or classes of service use [53]. LCA is a probabilistic model whereby for each participant the posterior probability of membership in each class is computed. Given the large sample size, even small likelihood-ratio (LR) chi-square values tend to be significant (indicating departure of observations from the model). Thus, we used common model fit criteria (i.e., Bayesian Information Criteria, BIC; Akaike Information Criteria, AIC; Consistent Akaike Information Criterion, CAIC) to determine the optimal number of classes [54, 55]. Given the sample sizes, small changes in the model fit might be suggested; thus, models ranging from 2 to 10 classes were applied to the data (e.g., [56]). Given that there are no established guidelines for what constitutes a substantive improvement in model fit for LCA, we used an a priori criterion to consider models with improvements in fit of 2% or more (averaging across the three fit criteria) over previous models to determine the optimal number of classes; we used this same criterion in our previous manuscript [27]. The program was set to start computations at 10 random points, to minimize the likelihood of deriving an unrepresentative local solution. For each solution, we specified 250 Expectation-Maximization iterations, followed by 50 Newton-Raphson iterations to optimize the class allocation [53]. A total of 500 bootstrap replications were computed. Visit descriptives for the different class solutions were reviewed with respect to substantive implications for understanding service use. Based on our previous study, it was hypothesized that five patterns of service use would be identified [27]. After selecting the number of classes, the contribution of the three client cohorts (defined by the calendar year of their first recorded visit; 2000, − 01, − 02), or the five CYMHS agencies in multi-level LCA was examined. The inclusion of these factors did not substantively improve the model fit; thus, the final LCA model did not include cohort or agency. The entropy value, an indicator of the certainty of classification of cases into classes, is reported; entropy values approaching 1.0 indicate clear delineation of classes [57]. Using probabilities estimated from the model, each client was allocated to a latent class [58].

#### Service use characteristics

Service use characteristics by pattern are reported. Differences in these variables between patterns of service use were examined using chi-square analysis or ANOVA, with Bonferroni post-hoc comparisons as applicable.

#### Predicting patterns of service use

Discriminant function analysis (DFA) was used to explore whether pattern of service use could be distinguished based on a combination of variables, including demographic factors (e.g., sex, age at first visit) and measures of psychopathology, child impairment, and family burden (i.e., BCFPI composite scales). Analyses were conducted in SPSS v24 [52]. A priori probabilities of assignment to patterns in classification were used. Wilks’ lambda is a ratio of the error variance to the pooled variance plus error variance and is the preferred statistic to measure model fit in DFA [59]; it is used to determine whether functions are significant, based on improvement in model fit (i.e., decreased values). Cross-validation was done using a jackknife, or leave-one-out, procedure within the Discriminate procedure [60, 61].

#### Descriptive analyses for predictors of service use

Descriptive statistics for predictor variables by pattern are reported. Again, differences in these variables between patterns of service use were examined using chi-square analysis or ANOVA, with Bonferroni post-hoc comparisons as applicable.

## Results

The final sample included 5632 children (62.3% male) whose age (years) at the time of their first visit was: 5 (8%), 6 (10%), 7 (9%), 8 (11%), 9 (12%), 10 (12%), 11 (12%), 12 (13%), or 13 (14%).

### Examining patterns of service use: latent class analysis (LCA)

Model fit improved in the LCA with the addition of each class from 2 to 10; however, the percentage improvement in the fit indices was less than 2% for models with more than 5 classes. Thus, the 5-class model was retained (see Additional file 1: Table S1). Multi-level modelling with the inclusion of cohort year (i.e., year of first visit – 2004, − 05, − 06) and agency did not result in improvements in the model fit. Thus, all analyses are reported collapsing across agencies and cohort years.

**Fig. 1:**
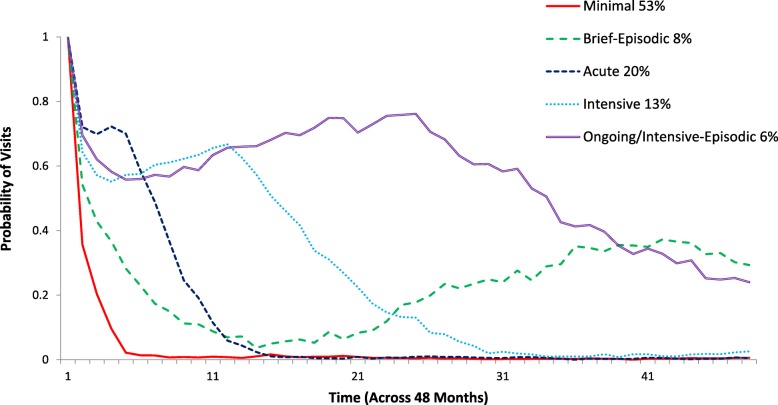
Patterns of service use based on five child and youth mental health service agencies across 4 years. The y-axis shows the probability of a child having at least one visit in each month. The x-axis shows months, starting with month 1 (the child’s first visit at the agency) through to the end of the observation period (4 years after the child’s first visit). For example, at month 11 a child in the Ongoing/Intensive-Episodic class had a 0.63 probability of a visit; in other words, 63% of this group of children were seen in month 11. At month 21, a child in the Intensive group had a 0.22 probability of being seen, whereas a child in the Brief Episodic group had only a 0.08 probability of being seen. Classes were labelled based on examination of the probability of visits over time and descriptive statistics on the overall duration of involvement and number of visits, along with the number and distribution of episodes of care (see Table 1 and Additional file 1)

Figure 1 shows the probability of visits over 4 years by classes. The classes were labelled based on the probability of visits over time and the descriptive data on duration of involvement, number of visits, and EoCs (see Table 1): Minimal (53.2% of cases); Brief Episodic (7.9%); Acute (20.1%); Intensive (13%); and Ongoing/ Intensive-Episodic (5.8%).

**Table 1:** Service Use and Demographic Characteristics by Pattern of Service Use

Pattern	N	Total	Sex^1^	Age^2^	Number of episodes			Duration of involvement (years)	Duration of involvement > 2 years	Visits over 4 years	Volume of all services^3^
			Male		0	1	2+				
		%	%	M (SD)	(%)	(%)	(%)	M (SD)	%	M (SD)	%
Minimal	2997	53	61.5	10.1 (2.6)^A^	60.9	37.3	1.8	0.4 (0.8)	6.3	3.1 (2.9)	9.8
Acute	1131	20	60.9	9.9 (2.5) ^AB^	***	95.6	4.2	0.8 (0.7)	8.2	15.6 (17.4)	18.5
Brief Episodic	447	8	62.2	9.3 (2.5) ^C^	***	27.3	72.0	3.5 (0.5)	99.8	28.6 (28.0)	14.2
Intensive	730	13	67.8	9.7 (2.5) ^B^	–	73.4	26.6	1.8 (0.8)	31.1	32.6 (28.6)	25.9
Ongoing/ Intensive-Episodic	327	6	61.2	9.9 (2.4) ^AB^	–	54.4	45.5	3.3 (0.6)	100.0	86.7 (105.8)	31.6
Total Sample	5632	100	62.3	9.9 (2.6)	32.5	53.9	13.7	1.1 (1.3)	22.8	16.3 (36.1)	100.0

*Note*. *** reflects < 5 children within the category; data not reported. --- reflects no cases in a cell

^1^Sex differed across patterns [χ^2^ (4) = 11.279, *p* = .024]. The Intensive pattern had proportionally more boys than expected [Adjusted residual χ^2^(1) = 11.022, *p* < .001]

^2^Age at intake differed across patterns [F (4, 5627) = 11.8, *p* < .0001]. Means followed by a common superscript letter are not significantly different at *p* < 0.05 (Bonferroni post-hoc test)

^3^Volume of all services: Visits were summed across all clients and all agencies within each of the five patterns, and divided by the sum of all visits for all clients and agencies

### Service use characteristics

Differences in service use characteristics were evident across the patterns (see Table 1; Additional file 1: Tables S2-S4 provide additional details on service use by EoCs). The number of EoCs [χ^2^(12) = 4125.04, *p* < 0.001] and duration of involvement varied across patterns [*F*(4, 5627) = 2676.81, *p* < 0.001; all pairwise comparisons *p* < 0.05]. Two groups tended to have multiple EoCs - the Brief and Ongoing/Intensive-Episodic patterns. Within the Brief Episodic group, close to one-third (31.8%) of children had, on average, 1.7 visits spread over 4.7 months before starting their first EoC 2 years later; during their first EoC they had 12.9 visits within about 6 months, followed by close to 2 years (23.3 months) without a visit, and then a second EoC of 18 visits over 8 months. Within the Ongoing/Intensive-Episodic group, only 10% of children had visits before starting their first EoC; during their first EoC they had 62.4 visits over about 2 years, which for 54% of cases was their only EoC. For the remaining 46% of cases, their second EoC occurred after 9 months without a visit, and included 48 visits over 16 months. Interestingly, across the entire sample, 7.5% of cases returned about 21 months after the last visit in their last episode of care for an average of 1.4 visits (see Additional file 1). Finally, with respect to the total volume of services delivered, the Ongoing/Intensive-Episodic group accounted for 32%, and the Intensive group 26%, of all visits for the entire sample (see Table 1).

### Predicting patterns of service use

Of the 5632 cases in the final sample, 3344 (59.4%) had a BCFPI. The percentage of cases having a BCFPI was virtually identical to the percentage of cases within each of the five patterns of service use (χ^2^ = 6.9, *p* = 0.139). Thus, the likelihood of a child having a BCFPI was not differentially related to future service use. Cases with a BCFPI were older (M = 10.2) than those without [M = 9.5; *t* (4107.16) = 9.958, *p* < 0.001], but did not significantly differ in terms of sex. Results from the DFA and descriptive analyses informed prediction of the patterns.

#### Discriminant function analysis (DFA)

Age, sex, and BCFPI composite scales were entered into a DFA. Two functions significantly discriminated between the five patterns, accounting for 94.5% of the explained variance (see Table 2). The overall chi-square test was significant, although with a high Wilks’ lambda (Wilks λ = 0.938, χ2 = 213.175, df = 24, *p* < 0.001), indicating that the model significantly predicts group members but a high proportion of variance is not accounted for by group membership [62]. Lambda improved when a second function was added (Wilks λ = 0.992, χ2 = 27.345, df = 15, *p* = 0.026; [62]). Classification of cases based on the new canonical variables correctly classified 51.6% of cases (51.4% in cross-validation).

**Table 2:** Predictors of Pattern of Service Use, Standardized Canonical Discriminant Function Coefficients and Structure Coefficients

	Function 1		Function 2	
	Family Burden, Externalizing, Impairment		Age, Internalizing	
Predictor	Standardized^a^	Structure^b^	Standardized^a^	Structure^b^
Sex	0.131	0.045	−0.031	−0.154
Age	−0.373	− 0.356	0.869	0.886
BCFPI Externalizing	0.377	0.791	0.007	0.239
BCFPI Internalizing	0.007	0.349	0.317	0.417
BCFPI Child Impairment	0.315	0.700	0.118	0.426
BCFPI Family Burden	0.416	0.816	0.137	0.296

*Note. BCFPI* Brief Child and Family Phone Interview

^a^Coefficients with larger absolute values reflect variables with greater discriminating ability

^b^Pooled within-groups correlations between predictor variables and standardized canonical discriminant function coefficients shown

**Fig. 2:**
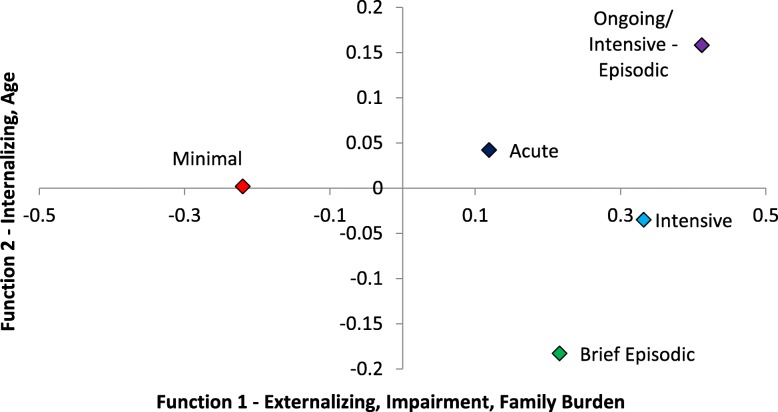
Patterns of service use group centroids on two functions based on discriminant function analysis. The standardized discriminant function coefficients and structure coefficients are reported in Table 2. Scores above zero on Function 1 reflect higher levels of externalizing, impairment (i.e., greater impairment), and family burden. Scores greater than zero on Function 2 reflect older age and higher internalizing scores. Tables 1 and 3 present descriptive statistics for age and the Brief Child and Family Phone Interview (BCFPI) variables by class

The first discriminant function (see Fig. 2 and Table 2) showed high Externalizing, Child Impairment, and Family Burden scores best differentiated the groups, while on the second function, primarily older age and, to a lesser degree, higher Internalizing scores did. Children in the Minimal pattern of service use were most distinguishable from those in the other groups as being lowest on Function 2 (reflecting lower Internalizing scores). The Brief Episodic group was most clearly differentiated by having the youngest children. The Acute and Intensive groups were similar on both functions. Finally, the Ongoing/Intensive-Episodic group had the highest mean scores on both functions, reflecting the highest levels of psychopathology, Child Impairment, and Family Burden.

#### Descriptive analyses by pattern

Age at intake differed across patterns [*F*(4, 5627) = 11.8, *p* < 0.0001; see Table 1]; children in the Brief Episodic service use pattern were younger than in all other patterns (post-hoc *p* < 0.05), and those in the Minimal pattern were significantly older than those in the Intensive pattern (*p* = 0.003). Sex was associated with pattern of service use [χ^2^(4) = 11.279, *p* = 0.024]; however, the only significant subgroup difference was that there were proportionally more boys in the Intensive pattern of service use than expected [Adjusted residual χ^2^(1) = 11.022, *p* < 0.001].

**Fig. 3:**
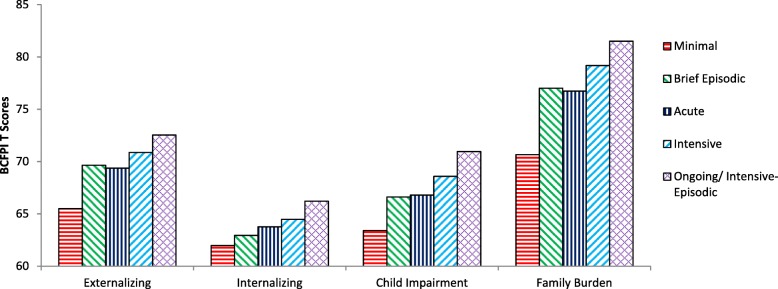
Average Brief Child and Family Phone Interview (BCFPI) composite scale scores by service use pattern

On the BCFPI, the Minimal pattern had significantly lower (*p* < 0.01) Externalizing, Child Impairment, and Family Burden scores than all other use patterns (see Fig. 3, Table 3). Children with this pattern also had significantly lower (*p* < 0.01) Internalizing scores than the Intensive and Ongoing/Intensive-Episodic patterns. Children in the Acute pattern had lower (*p* < 0.05) Externalizing scores than the Ongoing/Intensive-Episodic pattern. The Ongoing/Intensive-Episodic pattern had higher (*p* < 0.05) Child Impairment scores than all other patterns except the Intensive group, and higher Externalizing than the Minimal and Acute groups.

**Table 3:** Clinical Characteristics of Sample for Patterns of Service Use across the Five Child and Youth Mental Health Service Agencies

Clinical characteristics	Minimal	Acute	Brief Episodic	Intensive	Ongoing/Intensive-Episodic	Total
	n (%) or M (SD)	n (%) or M (SD)	n (%) or M (SD)	n (%) or M (SD)	n (%) or M (SD)	n (%) or M (SD)
Cases with BCFPI^1^ at intake (row %)	1719 (51.4%)	695 (20.8%)	257 (7.7%)	476 (14.2%)	198 (5.9%)	3344 (59.4%)
BCPFI at Intake^2^						
Externalizing M (SD)	65.5 (13.3)^A^	69.4 (12.9)^B^	69.6 (12.0)^BC^	70.9 (13.1)^BD^	72.5 (13.0)^CD^	67.8 (13.3)
Internalizing M (SD)	62.0 (14.0)^A^	63.8 (14.4)^AB^	63.0 (14.3)^AC^	64.5 (14.4)^BCD^	66.2 (15.3)^BCD^	63.0 (14.3)
Child Impairment M(SD)	63.4 (13.8)^A^	66.8 (13.9)^B^	66.6 (14.8)^BC^	68.6 (15.2)^BCD^	71.0 (15.2)^D^	65.6 (14.4)
Family Burden M (SD)	70.7 (19.3)^A^	76.7 (21.5)^B^	77.0 (20.6)^BC^	79.2 (21.6)^BCD^	81.5 (23.3)^BCD^	74.4 (20.8)
Externalizing T ≥ 65^3^	881 (51.3%)	434 (62.5%)	167 (65.0%)	323 (67.9%)	138 (69.7%)	1943 (58.1%)
Internalizing T ≥ 65^3^	632 (36.8%)	280 (40.3%)	99 (38.5%)	209 (43.9%)	95 (48.0%)	1315 (39.3%)
Child Impairment T ≥ 65^3^	758 (44.1%)	373 (53.7%)	143 (55.6%)	267 (56.1%)	126 (63.6%)	1667 (49.9%)
Family Burden T ≥ 65^3^	825 (48.0%)	429 (61.8%)	164 (63.8%)	313 (65.8%)	134 (67.7%)	1865 (55.8%)
Comorbidity at Intake						
Sub-clinical levels (i.e., T < 65%)	624 (36.3%)	178 (25.6%)	65 (25.3%)	97 (20.4%)	37 (18.7%)	1001 (29.9%)
Externalizing or Internalizing T ≥ 65^3^	659 (38.3%)	313 (45.1%)	112 (43.6%)	224 (47.1%)	89 (44.9%)	1397 (41.8%)
Externalizing and Internalizing T ≥ 65^3^	425 (24.7%)	200 (28.8%)	76 (29.6%)	154 (32.4%)	72 (36.4%)	927 (27.7%)

*Note*. % = Column percentages are reported, except as noted for cases with BCFPI

^1^*BCFPI* Brief Child and Family Phone Interview

^2^For each row variable, means followed by a common superscript letter are not significantly different at *p* < 0.05 (Bonferroni post-hoc test). Frequencies are a percentage of subsample with BCFPI present

^3^T-scores computed from age- and sex-based population norms

## Discussion

Five patterns of services use emerged from visit data for 5–13 year olds first seen in 2004, − 05, or − 06 at one of five CYMHS agencies in Ontario. Thus, for the first aim of the study we replicated five patterns of service use that were virtually identical to a previous study [27], which included cohorts of children first seen in 2000–02. The percent of cases classified into each pattern varied by only 3% (Additional file 1: Table S5 presents descriptive statistics for the two studies). Differences may be due to a shorter study duration (4 years vs 5), or a secular trend towards briefer treatment.

The second aim of the study was to examine predictors of patterns of service use. The accuracy in predicting children’s pattern of service use based on their demographic information (age) and BCFPI scores (Internalizing, Externalizing, Child Impairment, Family Burden) at the time of their first visit was just over 50%. Lower levels of Externalizing, Child Impairment, and Family Burden differentiated the Minimal care pattern from all others. In contrast, the Ongoing/Intensive-Episodic pattern had the highest levels on the BCFPI variables, with Child Impairment being significantly higher than all other patterns except the Intensive group, and higher Externalizing than the Minimal and Acute groups. There were few differences between the Brief-Episodic, Acute, and Intensive groups.

Some findings in the current study were not surprising. For example, in the Acute pattern, children tended to be seen for one EoC lasting about 10 months with an average of about 16 visits. Although we were unable to document the type of treatment children received, this pattern was the most aligned to the provision of an EBT protocol. For comparison, in a US study where agency staff in community CYMHS agencies delivered two EBTs, clients received an average of 16 treatment sessions delivered over 196–210 days [63].

We first discuss the Minimal care pattern, as it reflects 50% of all cases. We then focus on episodic vs ongoing service use, as little is known about how to best care for children receiving services in these ways. Findings related to predicting patterns are discussed, where relevant. We conclude with a discussion of limitations and over-arching implications for CYMHS.

### Minimal vs episodic vs ongoing service use

#### Minimal care

Over 50% of cases were seen for only a few visits. These results are very similar to other studies. For example, analyses of US private health insurance data for child and youth mental health services found that 45% of cases were seen for less than 1 month, and 78% for less than 6 months [25]. In the Fort Bragg study, 61% of cases were treated for less than 6 months [64]. In a community-based prospective survey, 47–51% of children and youth had fewer than four counselling visits with a mental health specialist [46]. Private insurance might place limits on treatment duration [25, 46], resulting in a large number of cases with short treatment durations. This was not true in the Fort Bragg study [65], nor is it true in the Ontario system.

It is not clear whether shorter treatment durations and/or few sessions reflect inadequate care, as has been suggested by some authors. For example, Saloner et al. [46] used eight sessions as the minimum number of mental health visits. Eight sessions has been recommended as the minimum for adolescent depression, but the authors noted that “no meta-analyses of brief counseling treatments exist for adolescent populations” ([66], p. 100). Contrary data are provided in another treatment study for adolescents with depression; 31% of cases demonstrated substantive improvement (> 50%) by the second treatment session, without increased risk of poor outcomes, up to 2 years post treatment [67].

Minimal service use patterns may reflect well-known problems with client engagement in CYMHS [68–70]. However, with more than half of all children falling into the Minimal pattern, this finding also supports the need for brief intervention services and walk-in clinics [71–73]. In Ontario’s most recent child mental health treatment plan [74], brief services are one of seven core services to be available at all CYMHS agencies in the province. Children in the Minimal service use pattern had the lowest level of family burden, child impairment, and externalizing behaviors, and the fact that few children/families in this pattern returned for care in the following 4 years, suggests that just a few appointments may be sufficient to meet the needs of these families. Thus, the label “Minimal” should not be interpreted to mean inadequate or inappropriate care. Rather, it captures the relative number of visits and duration of involvement of this group of children compared to the other four patterns of service use. Research and practice would benefit from ongoing outcome monitoring and targeted studies on brief services to help inform our understanding of the appropriateness of brief treatments in CYMHS [75, 76].

#### Episodic service use

In this and our previous study, 15–20% of children were seen for more than one EoC within a 4–5 year period [27]. The Brief Episodic pattern of service had the highest percentage of children having two or more EoCs. Clinically, they were similar to children in the Acute and Intensive service use groups, as family burden, child impairment, and externalizing behaviors did not differentially predict these three patterns.

Only one other study reported similar data on EoCs. Warren et al. [77] analyzed administrative data from 1997 to 2008 for 4–17 year olds seen in either a community mental health agency (*N* = 3524) or a managed care setting. They reported that the average number of EoCs was 1.9 in the community agency. EoCs were, on average, 9.5 weeks long and consisted of 2.9 sessions. However, this study had a markedly different definition of an EoC; Warren et al. defined an EoC as 90 days without a visit, which presumably meant that an EoC could be only one visit, whereas we defined an EoC as a minimum of three visits with 180 days with no visits in between episodes [77]. There have been other studies that discuss EoCs, but again, data are not directly comparable. These studies focus on specific diagnostic groups (e.g., depression [78, 79]), medication use (e.g., for ADHD [80]), used much shorter time frames (e.g., 180 days, [25]), or combined health and mental health services [e.g., [46]].

What are the reasons that children receive more than one EoC? Sytema et al. [81] suggested that patterns of care “reflect both the functioning of mental health care system and the help-seeking behavior of its clients” (p. 1). The natural history of anxiety and depression is that many children re-experience these problems. In both community and clinical samples, 50–70% of youth with depression will experience another episode within 3 years; anxiety disorders also tend to be episodic [3]. If children experience disorders as waxing and waning over time, we would expect that experiencing more than one episode of an illness would lead to more than one EoC. Problems such as ADHD have been conceptualized as chronic, but treatment is often episodic. In a 7-year, population-based study of children and youth prescribed methylphenidate, one-third of cases had more than one episode (i.e., no prescriptions being filled for 4 months) of treatment, with 10.2% of cases having three or more episodes [80]. This suggests that some EoCs are due to the natural history of the underlying condition, whereas others occur due to variation in family help-seeking patterns. Episodic service use could also be due to dropout; this will be explored in a future article.

#### Ongoing service use

A sizeable proportion of children were seen many times; children in the Intensive and Ongoing/Intensive-Episodic patterns were seen, on average, for 33 and 87 visits, respectively. Virtually all cases (27% of the sample) within three of the service use patterns (Brief Episodic, Intensive, and Ongoing/Intensive-Episodic) had involvement for more than a year, and 24% of all cases were seen for more than 2 years.

Although there are no studies that provide directly comparable data, three studies report service use over longer time periods. Mueller et al. [82] analyzed data from children with severe mental and behavioral disorders treated within the publicly-funded system in Hawaii. After excluding all cases seen for fewer than 90 days, the average duration of a “service episode” was about one and a half years (560.2 days; SD = 372.2). While it was unclear how a service episode was defined, their data are similar to the current study in finding that many children are seen for longer than a year, and that there is considerable variability in durations of care. A 2-year follow-up from a randomized clinical trial conducted in community agencies in the US testing usual care versus standard EBTs versus modular treatment found that 13% (standard EBT) to 18% (modular) of cases received treatment from a community mental health clinic from 1 to 2 years after the trial ended. Finally, Goldstein et al. [13] reported that 50–60% of children who had anxiety or depression had “long” durations of treatment (“continuous treatment of several years or numerous brief periods”; p. 970) when assessed as young adults. Collectively, our data and those from these studies demonstrate that a sizeable percentage of children and youth are seen for years, not months, and have considerably more treatment sessions than any EBT protocol. The sector needs to consider how to best care for children whose needs extend over longer periods of time, the implications of their care in other sectors (e.g., health), and what happens to these children when they transition to adulthood.

Of note, children who were seen for the longest period of time and had the highest number of visits (Ongoing/Intensive-Episodic pattern) were characterized as also having the highest severity of problems, particularly in terms of externalizing behaviors and level of impairment; parents of these children also reported the highest level of burden/impact on their family. Thus, the long duration and high volume of sessions might be appropriate. While representing only 6% of cases, children in this pattern had 32% of all visits by volume over the 4-year study period. If these children could be adequately cared for while making modest reductions in total visits, this could free resources to manage other demands, such as reducing waiting times.

### Recommendations for new approaches to CYMH care

Two key recommendations for CYMHS emerge from our findings. First, we recommend a triage approach, based on client characteristics at intake, be combined with a stepped-care approach. Triage is recommended to identify children and families at the extremes of need. Children with the lowest severity problems (i.e., the Minimal care pattern) could be directed immediately for single session or walk-in services. Children/families with the highest level of problems are most likely to need ongoing care; these cases are unlikely to benefit from brief interventions, which is the typical starting point in stepped care models. Children in the Ongoing/Intensive-Episodic pattern had the highest level of externalizing problems and impairment, and their parents reported very high levels of burden. These cases should be triaged into intensive services as soon as possible; this might include case management along with individual and parent treatment.

For children whose problems are neither very mild nor severe, a stepped-care model would be appropriate. This would be the Acute, Intensive, and Brief Episodic patterns in our sample, for whom the clinical characteristics at intake did not differ markedly. In stepped-care models [83], children first receive a low-intensity treatment (e.g., self-help CBT via internet) and if this treatment is not effective, more intensive and/or additional treatments are added (e.g., CBT delivered in-person, medication; [84, 85]).

Second, the sector needs to develop different ways to care for children over extended periods of time. A recent article suggested using a life-span perspective and argued for applying the idea of a “health home” for coordination of care for children and youth with mental health problems [86]. These authors propose an integration of various delivery models, including the chronic care model [87, 88], systems of care [89], wraparound [90], and the Institute of Medicine mental health intervention spectrum [91, 92]. A shortcoming of their proposal is that they appear to suggest that their model be applied to all children and youth with mental health problems. We maintain that this is not feasible, given ongoing, unmet demands for CYMHS. A chronic care approach may be appropriate. Children with chronic physical health problems receive regular follow-up appointments and adjustments to treatment plans are made as needed [93, 94]. By having regularly scheduled follow-up appointments that involve monitoring of clients’ health and refinement of treatment plans, relapse might be reduced and health status maintained or improved. Such a model might be appropriate to implement for children seen for extended periods of time (e.g., > 2 years) or repeated EoCs, which would include about a quarter of children in our sample. The specific elements in such a model need to be determined, as would the markers of when to shift away from an “acute” treatment approach. We also do not know what children and parents might do to maintain treatment gains following an acute treatment phase or after discharge, for children who are likely to need a second EoC. Understanding positive mental health behaviors should be explored in future studies.

### Limitations

Analyses captured data only from participating CYMHS agencies. MH services provided in other sectors were not included and we know that children seen at one agency may be seen at other CYMHS agencies and other sectors [95, 96]. Examining service use over multiple years and across sectors is an important, but challenging, next step for future research. We recently linked the data from the current study to these children’s health care utilization data [97]. We are currently examining relationships between receipt of mental health services from physicians before, during, and after when children were receiving services from a children’s mental health agency [98], and predictors of receiving mental health services from physicians as young adults [99]. The service use patterns identified replicate those found in our previous work [27]. However, we cannot be certain that these patterns would be the same in all CYMHS agencies in Ontario or other jurisdictions.

The administrative data used was lacking a number of variables that could differentiate the patterns of service use such as single parent status, child welfare involvement, family socio-economic status, and ethnicity (e.g., [31, 100]). Future research might also consider agency-specific factors such as hours of operation and distance from clients’ homes, as ease of accessibility would also likely influence service use.

These analyses do not capture important elements related to families’ use of services. For example, we did not examine dropout or changes in outcomes. Dropping out of services and the magnitude of change in outcomes are both important variables that would likely impact service use over time [101, 102], as well as seeking help for a second time at the same agency [103], at other agencies and/or in other sectors.

## Conclusions

Examining and understanding patterns of service use within CYMHS and other sectors is critical before tackling the development and testing of new treatment models. By using existing data routinely collected at agencies, data on service use patterns can be directly applied in ways that can: (a) help agencies refine service delivery systems to better meet the needs of their client populations; (b) better allocate resources in response to the needs of their client populations; and (c) start a process of developing new models of service delivery.

## Supplementary information

**Additional file 1: Table S1.** Model Fit Indices for Testing 2–10 Class Solutions for Patterns of Service Use across Five Child and Youth Mental Health Service Agencies. **Table S2.** Mean Number of Visits in Each Period of Service Involvement across 4 Years by Service Use Pattern. **Table S3.** Breakdown of Duration of Involvement (in Months) across 4 Years by Service Use Pattern. **Table S4.** Distribution of Cases across Episodes of Care over 4 Years by Service Use Pattern. **Table S5. A** Sample Size and Number of Episodes by Pattern of Service Use in the Previous Study and the Present Study. **B** Service Use Descriptive Statistics by Pattern of Service Use in the Previous Study and the Present Study.

## Data Availability

The data that support the findings of this study are available from five CYMHS agencies in Ontario, Canada, but restrictions apply to the availability of these data, which were used under license for the current study, and so are not publicly available. Data are however available from the authors upon reasonable request and with permission of the CYMHS agencies, and in accordance with research ethics board policies.

## Abbreviations

AIC: Akaike information criteria
BCFPI: Brief child and family phone interview
BIC: Bayesian information criteria
CAIC: Consistent Akaike information criterion
CBT: Cognitive behavioral therapy
CYMHS: Child and youth mental health services
DFA: Discriminant function analysis
EBT: Evidence-based treatment
EoC: Episode of care
LCA: Latent class analysis
LR: Likelihood-ratio
MH: Mental health
