# Supplementary material for: Predicting patterns of service utilization within children’s mental health agencies
Source: BMC Health Serv Res. 2019 Dec 23;19:993. doi: 10.1186/s12913-019-4842-2 (PMC6929287; doi:10.1186/s12913-019-4842-2)
Supplement: Supplementary file 1 — Additional file 1: Table S1. Model Fit Indices for Testing 2–10 Class Solutions for Patterns of Service Use across Five Child and Youth Mental Health Service Agencies. Table S2. Mean Number of Visits in Each Period of Service Involvement across 4 Years by Service Use Pattern. Table S3. Breakdown of Duration of Involvement (in Months) across 4 Years by Service Use Pattern. Table S4. Distribution of Cases across Episodes of Care over 4 Years by Service Use Pattern. Table S5. A Sample Size and Number of Episodes by Pattern of Service Use in the Previous Study and the Present Study. B Service Use Descriptive Statistics by Pattern of Service Use in the Previous Study and the Present Study. [file 12913_2019_4842_MOESM1_ESM.docx]

**Additional file 1**

Table S1

*Model Fit Indices for Testing 2-10 Class Solutions for Patterns of Service Use across Five Child and Youth Mental Health Service Agencies*

| Number of Classes | Model fit indices | | | | | | | |
| --- | --- | --- | --- | --- | --- | --- | --- | --- |
|  | BIC | BIC  % Diff | AIC | AIC  % Diff | CAIC | CAIC  % Diff | LL | -2LL Diff |
| 2 | 134618 |  | 133987 |  | 134082 |  |  |  |
| 3 | 125123 | 7.05% | 124174 | 7.32% | 124317 | 7.28% | -61944.18 | 9908.76 |
| 4 | 120046 | 4.06% | 118779 | 4.35% | 118970 | 4.30% | -59198.42 | 5491.52 |
| 5 | 116430 | 3.01% | 114844 | 3.31% | 115083 | 3.27% | -57182.86 | 4031.14 |
| 6 | 114372 | 1.77% | 112467 | 2.07% | 112754 | 2.02% | -55946.67 | 2472.38 |
| 7 | 112861 | 1.32% | 110637 | 1.63% | 110972 | 1.58% | -54983.69 | 1925.94 |
| 8 | 112067 | 0.70% | 109525 | 1.01% | 109908 | 0.96% | -54379.53 | 1208.33 |
| 9 | 111448 | 0.55% | 108587 | 0.86% | 109018 | 0.81% | -53862.70 | 1033.67 |
| 10 | 110890 | 0.50% | 107711 | 0.81% | 108190 | 0.76% | -53376.63 | 972.12 |

*Note*. BIC = Bayesian Information Criteria; AIC = Akaike Information Criteria; CAIC = Consistent Akaike Information Criterion; % Diff = percentage change in model fit index with addition of 1 class; LL = Log-Likelihood; -2LL Diff = Bootstrap -2 log likelihood difference test.

Table S2

*Mean Number of Visits in Each Period of Service Involvement across 4 Years by Service Use Pattern*

| Pattern | Pre EoC^1^ | EoC 1 | EoC 1-2  Interval^2^ | EoC 2 | EoC 2-3  Interval | EoC 3 | Post EoC^1^ |
| --- | --- | --- | --- | --- | --- | --- | --- |
|  | M (SD) | M (SD) | M (SD) | M (SD) | M (SD) | M (SD) | M (SD) |
| Minimal | 1.5 (0.6) | 5.3 (2.8) | *** | 4.6 (1.9) | --- | --- | 1.4 (0.5) |
| Acute | 1.8 (0.9) | 15.2 (17.4) | *** | 5.2 (3.3) | --- | --- | 1.5 (0.6) |
| Brief Episodic | 1.7 (0.9) | 12.9 (18.7) | 1.3 (0.5) | 18.0 (23.4) | *** | 17.4 (20.1) | 1.7 (0.9) |
| Intensive | 1.4 (0.5) | 28.4 (27.0) | *** | 14.0 (26.6) | *** | 6.0 (1.6) | 1.3 (0.5) |
| Ongoing/Intensive-Episodic | 1.3 (0.5) | 62.4 (78.7) | *** | 47.5 (110.8) | *** | 30.4 (76.0) | 1.5 (0.5) |
|  |  |  |  |  |  |  |  |
| Total Sample | 1.5 (0.7) | 18.5 (32.4) | 1.4 (0.5) | 21.0 (54.5) | 1.4 (0.5) | 19.9 (43.9) | 1.4 (0.6) |

*Note*. EoC = Episode of Care, defined as at least 3 visits clustered within a 180 day period.

*** reflects < 5 children within the category; data not reported. --- reflects no cases in a cell; < 5 children had a fourth EoC; data not depicted.

**^1^** Pre-EoC reflects visits that occurred before an EoC, or all visits when children had < 3 visits overall. Post-EoC refers to visits that occurred after the child’s last EoC.

**^2^** EoC 1-2 Interval refers to visits that occurred in between the child’s first and second EoC.

Table S3

*Breakdown of Duration of Involvement (in Months) across 4 Years by Service Use Pattern*

| Pattern | Pre  EoC | Pre EoC-EoC1  Interval | EoC 1 | EoC 1-2  Interval | EoC 2 | EoC 2-3  Interval | EoC 3 | EoC -  Post EoC  Interval | Post  EoC |  | Overall^1^ |
| --- | --- | --- | --- | --- | --- | --- | --- | --- | --- | --- | --- |
|  | M (SD) | M (SD) | M (SD) | M (SD) | M (SD) | M (SD) | M (SD) | M (SD) | M (SD) |  | M (SD) |
| Minimal | 2.7 (8.3) | 17.8 (11.3) | 2.1 (1.3) | 18.2 (10.4) | 2.0 (1.7) | --- | --- | 23.4 (12.8) | 1.2 (4.1) |  | 4.4 (9.7) |
| Acute | 2.1 (5.5) | 7.1 (0.8) | 6.8 (2.5) | 16.6 (9.3) | 2.1 (1.5) | --- | --- | 18.8 (10.6) | 1.7 (4.2) |  | 9.9 (8.6) |
| Brief Episodic | 4.7 (9.5) | 23.8 (9.8) | 6.2 (4.8) | 23.3 (9.6) | 8.1 (6.3) | 13.1 (6.5) | 8.1 (6.3) | 26.6 (11.3) | 2.3 (4.2) |  | 42.4 (8.1) |
| Intensive | 0.9 (2.1) | 9.1 (2.5) | 13.4 (6.8) | 11.1(6.1) | 5.6 (3.5) | 18.7 (5.7) | 2.7 (0.7) | 18.3 (10.3) | 1.7 (4.7) |  | 22.3 (10.3) |
| Ongoing/ Intensive-Episodic | 0.2 (0.4) | 11.2 (2.9) | 24.6 (14.4) | 9.2 (3.0) | 16.0 (10.0) | 9.8 (3.6) | 7.4 (5.9) | 16.8 (11.4) | 1.6 (2.7) |  | 40.0 (8.1) |
|  |  |  |  |  |  |  |  |  |  |  |  |
| Total Sample | 2.7 (8.1) | 16.7 (10.4) | 8.1 (8.5) | 16.7 (10.0) | 8.2 (7.6) | 12.8 (6.2) | 7.3 (5.9) | 20.7 (11.7) | 1.6 (4.2) |  | 12.9 (15.9) |

*Note*: EoC = Episode of Care, defined as at least 3 visits clustered within a 180 day period. Pre-EoC reflect visits that occurred either prior to an EoC, or all visits when children had < 3 visits overall.

--- reflects no cases in a cell; < 5 children had a fourth EoC and are not depicted here.

^1^ Overall – Duration of involvement reflecting the difference between date of first and last face-to-face visits across 4 year study period.

Table S4

*Distribution of Cases across Episodes of Care over 4 Years by Service Use Pattern*

| Pattern |  | Pre EoC | EoC 1 | EoC 1-2  Interval | EoC 2 | EoC 2-3  Interval | EoC 3 | Post EoC |
| --- | --- | --- | --- | --- | --- | --- | --- | --- |
|  | N | Row % | Row % | Row % | Row % | Row % | Row % | Row % |
| Minimal | 2997 | 63.1 | 39.1 | *** | 1.8 | --- | --- | 4.5 |
| Acute | 1131 | 1.9 | 99.7 | *** | 4.2 | --- | --- | 11.0 |
| Brief Episodic | 447 | 31.8 | 99.3 | 7.8 | 72.0 | *** | 11.4 | 7.8 |
| Intensive | 730 | 12.3 | 100.0 | *** | 26.6 | *** | 1.4 | 14.7 |
| Ongoing/Intensive-Episodic | 327 | 11.0 | 100.0 | *** | 45.6 | *** | 7.6 | 7.0 |
|  |  |  |  |  |  |  |  |  |
| Total Sample | 5632 | 38.7 | 67.5 | 0.8 | 13.7 | 0.1 | 1.5 | 7.5 |

*Note*. EoC = Episode of Care, defined as at least 3 visits clustered within a 180 day period. *** reflects < 5 children within the category; data not reported. --- reflects no cases in a cell; < 5 children had a fourth EoC and are not depicted here.

Row % reflects the percentage of cases within each pattern that had one or more visits before an EoC (pre EoC), in the time period between EoCs (EoC 1-2 Interval, EoC 2-3 Interval) or after the final EoC (Post EoC); also reported are the percentages that had an EoC (EoC1, EoC2, EoC3).

Table S5. A

*Sample Size and Number of Episodes by Pattern of Service Use in the Previous Study and the Present Study*

| Study^1^ | A | | B | | A | | | B | | |
| --- | --- | --- | --- | --- | --- | --- | --- | --- | --- | --- |
| Pattern | N | Total | N | Total | Number of episodes | | | Number of episodes | | |
|  |  |  |  |  | 0 | 1 | 2+ | 0 | 1 | 2+ |
|  |  | % |  | % | (%) | (%) | (%) | (%) | (%) | (%) |
| Minimal | 3796 | 50 | 2997 | 53 | 37.9 | 58.0 | 4.1 | 60.9 | 37.3 | 1.8 |
| Acute | 869 | 21 | 1131 | 20 | 7.1 | 85.7 | 7.3 | *** | 95.6 | 4.2 |
| Brief Episodic | 1610 | 12 | 447 | 8 | *** | 21.2 | 77.3 | *** | 27.3 | 72.0 |
| Intensive | 933 | 11 | 730 | 13 | *** | 70.9 | 28.7 | ---- | 73.4 | 26.6 |
| Ongoing/ Intensive-Episodic | 430 | 6 | 327 | 6 | ---- | 44.9 | 55.2 | ---- | 54.4 | 45.5 |
|  |  |  |  |  |  |  |  |  |  |  |
| Total | 7638 | 100 | 5632 | 100 | 20.6 | 60.1 | 19.4 | 32.5 | 53.9 | 13.7 |

*Note*. *** reflects < 25 children within the category; data not reported. --- reflects no cases in a cell. Percentages refer to percentage of cases of respective study.

^1^ Study A = Previous study (2000, -01, or -02 cohort); Study B = Present study (2004, -05, or -06 cohort).

Table S5. B

*Service Use Descriptive Statistics by Pattern of Service Use in the Previous Study and the Present Study*

| Study^1^ | A | B | A | B | A | B | A | B |
| --- | --- | --- | --- | --- | --- | --- | --- | --- |
| Pattern | Duration of involvement  (years) | Duration of involvement  (years) | Duration of involvement  >2 years | Duration of involvement  >2 years | Visits  over 4 years | Visits  over 4 years | Volume of all services^2^ | Volume of all services^2^ |
|  | M (SD) | M (SD) | % | % | M (SD) | M (SD) | % | % |
| Minimal | 0.4 (0.8) | 0.4 (0.8) | 4.7 | 6.3 | 5.6 (5.3) | 3.1 (2.9) | 15.6 | 9.8 |
| Acute | 1.0 (0.8) | 0.8 (0.7) | 6.5 | 8.2 | 18.4 (14.4) | 15.6 (17.4) | 18.0 | 18.5 |
| Brief Episodic | 3.7 (0.9) | 3.5 (0.5) | 98.8 | 99.8 | 25.4 (17.0) | 28.6 (28.0) | 15.0 | 14.2 |
| Intensive | 2.3 (0.9) | 1.8 (0.8) | 51.9 | 31.1 | 47.3 (32.4) | 32.6 (28.6) | 24.5 | 25.9 |
| Ongoing/ Intensive-Episodic | 4.2 (0.7) | 3.3 (0.6) | 100 | 100.0 | 104.8 (58.9) | 86.7 (105.8) | 27.0 | 31.6 |
|  |  |  |  |  |  |  |  |  |
| Total | 1.4 (1.5) | 1.1 (1.3) | 27.3 | 22.8 | 21.1 (31.7) | 16.3 (36.1) | 100 | 100 |

*Note*. Percentages refer to percentage of cases of respective study.

^1^ Study A = Previous study (2000, -01, or -02 cohort); Study B = Present study (2004, -05, or -06 cohort).

^2^ Volume of all services: Visits were summed across all clients and all agencies within each of the five patterns, and divided by the sum of all visits for all clients and agencies.
